# Recamier on Cancer

**Published:** 1830-04-01

**Authors:** 


### XXII.

### On the Treatment of Cancer by Compression. By M. Recamier, M.D.

The talented physician above-mentioned has published two large volumes on a method of treatment which was first brought into vogue in this country, by Mr. Young, in 1816 or 1817, abandoned, after a trial, (whether fair or not we cannot say,) in the cancer ward of the Middlesex Hospital. Dr. Recamier says that the want of success in this country, was owing to the want of a proper mode of applying the pressure—and there may be something in this. At all events, the practical results of such a man as Recamier, should not be despised.

Of 45 cases of cancer of the breast, 30 were treated by simple compression alone—4 by compression and cauterization—5 by compression and ablation—and 6 by a combination of these three means. Of these 45 cases, 20 were cured—15 remain under cure, and a few of them promise success—ten have entirely failed. The greater number of the patients were females between the age of 30 and 60 years—and more especially between 40 and 50, namely at the turn of life. He met with only 1 case below the age of 12—and one above 70. Of the foregoing cases 11 were relapses after amputation of the breast before M. R. was consulted. One only relapsed after being considered cured by the author.

One case is mentioned by the author, of cancer of the stomach, which is deserving of notice. A female aged 55 years, had never had an illness of any consequence, except an erysipelas in her legs, which was several times reproduced. She ceased to menstruate at 45, and from that period experienced sad reverses of fortune, and great anxiety of mind. Next she became affected with pain in the region of the stomach, and frequent vomitings of dark-coloured stuff, resembling the lees of wine. Her features underwent a great change—her skin assumed the yellow and sallow tint—marasmus increased—and an oval tumour, transmitting the pulsations of the aorta, became established in the region of the pylorus. Solid food could not be taken at all, and liquids were obliged to be given in spoonfuls at a time, on account of the vomitings. All means of relief had failed, and the patient was reduced to the verge of the grave, when a girdle was constructed so as to make pressure on the tumour. This was in the beginning of November. In February following the patient was able to leave her bed, and take to her domestic avocations. The tumour was reduced in size, but the bandage continues to be worn. If this be a true representation, and the tumour be actually a scirrhus of the pylorus, the case is very curious.

In respect to the mode of applying pressure in cancer, much must, of course, depend on the manual dexterity of the applicant. Dr. Recamier tells us that the pressure must be perfectly equal over the whole surface. He has tried various substances, such as chamois leather, cotton, bladders distended with air, disks of India-rubber, &c. but nothing has yet succeeded so well as layers of agaric, cut smoothly, and applied over the swelling. These he retains on the part by bandages of flannel, without seams or selvidges, rather more than 2 inches in breadth, and eight or nine yards long. He places a disk of agaric on each breast, and then several additional layers on the one affected with scirrhus, locating them so that the centre of pressure may fall on the most prominent part of the tumour. When this last is very prominent, the disk of agaric must be thick in proportion, and vice versâ. The pressure of the bandage cannot be borne by some patients, unless it be very skilfully applied, and his art of application cannot be conveyed in words. It is the *lex non scripta* of surgery, and to the skill of the individual we must leave it.

In respect to the cauterization, M. Recamier employs the concentrated nitric acid as the agent, to each ounce of which he adds a drachm or less of the crystallized nitrate of quicksilver. Arsenic he considers to be dangerous, as he has seen several cases where its application has been followed by death with all the symptoms of poisoning by arsenic. The pain of the acid cauterization is mitigated by the application of pledgets of lint saturated with laudanum. Verjuice combined with opiates internally appeared to soothe the sufferings better than opium alone.

M. Recamier thinks that relapses of cancer are more common after simple ablation of the tumour, than when cauterization has been employed before the operation by the knife.

In so rebellious and fatal a malady as cancer, the observations of M. Recamier, who is a practical physician of very considerable genius and talents, should not be scouted. We know indeed that the remedy proposed by him has failed in this country, when Mr. Young's method was put to the proof in a public institution; but still we know that other remedies have suffered a similar fate, and been subsequently found deserving of a better one.

And here we may remark a passage in Mr. Bell's report from the Middlesex Hospital, on the inefficacy of compression. The Medical Committee then stated,

"That they had, *in many cases*, succeeded in obtaining great alleviation of suffering—such alleviation as might, perhaps, induce more speculative minds less disciplined by experience, to conclude that they had at length succeeded in discovering a cure for cancer."*

Now as Mr. Bell has not, in that report, let fall the least hint of what these means of alleviation (amounting *almost to a cure* of cancer) consisted; and as we cannot charge our memory with any subsequent annunciation of such gratifying information, we do call upon that distinguished surgeon, who, we know well, detests any thing like quackery, to say what those means were, and whether fourteen years of additional experience has confirmed or dissipated the hopes which might naturally be grounded on such a notice, and from such a source.

---

* First quarterly report of cases in surgery. By Mr. Charles Bell, 1816.

